# Self-Hierarchy in Perceptual Matching: Variations in Different Processing Stages

**DOI:** 10.3389/fpsyg.2022.770604

**Published:** 2022-04-06

**Authors:** Yingcan Zheng, Zilun Xiao, Yong Liu, Xin Zhou

**Affiliations:** ^1^Developmental Psychology for Armyman, Department of Medical Psychology, Army Medical University, Chongqing, China; ^2^School of Psychology, Southwest University, Chongqing, China

**Keywords:** self-hierarchy, collectivism, self-construct, situational, perceptual matching, ERP

## Abstract

People have three cognitive representations of the self, namely, the individual, relational, and collective selves (CS), which are indispensable components of selfhood but not necessarily given equal preference. Previous studies found that people displayed varied self-hierarchy in miscellaneous tasks involving different research materials that had pre-existing learned associations established over long periods of time. Therefore, this study tries to explore a purer self-hierarchy without the influence of research materials, using perceptual matching tasks. The behavioral and event-related potentials’ (ERPs) findings showed that people recognized information association with their individual self (IS) faster compared with their relational (RS) and CS. Smaller N2, stronger P3 and late positive complex (LPC) amplitudes were evoked during IS compared with RS and CS. However, the three selves evoked equal P2 amplitudes at the early processing stage. Moreover, CS showed a weaker advantage than RS, demonstrating a longer reaction time, lower d prime, and weaker P3 and LPC amplitudes in the parietal region. Overall, self-hierarchy during simple perceptual processing manifested as IS > RS > CS at the late processing stage but manifested as IS = RS = CS at the early processing stage. Self-hierarchy varies according to the processing stage, even without meaningful information and during the simple perception processing. This result provides direct evidence that all selves can be tagged with neutral actions, which would fit the idea of an organism attuned to self-survival at multiple processing levels.

## Introduction

The tripartite model of self-construal theory indicated that the three selves have different hierarchies, and self-hierarchy will be affected by social situations. Previous studies found that people displayed varied self-hierarchy across different tasks. However, varied self-hierarchy may be largely influenced by the design of these studies. Therefore, this study attempts to explore a purer self-hierarchy without the influence of research materials.

The tripartite model of self-construal theory suggested that people have three cognitive representations of the self: the individual, relational, and collective self (Triandis, 1989; Brewer and Gardner, 1996). These three selves coexist in each human and all selves are important for people to meet needs such as securing inclusion in social situations, achievements, and happiness (Nehrlich et al., 2019; Long et al., 2020). However, there is a wide controversy on the comparative advantage of these three selves, based on self-hierarchy. As studies progressed, researchers confirmed that self-hierarchy depends on situational factors (Sedikides et al., 2013; Nehrlich et al., 2019). Studies on work self-concept, role theory, and the kaleidoscopic self show that self-representation changes with situational factors such as norm salience, role importance, and fleeting social circumstances (Gao and Wang, 2017). Thus, self-hierarchy has dynamic situational characteristics.

As per previous research, the situational nature of self-hierarchy is related to the different tasks chosen, which involve different processes. When researchers used the odd-ball task to investigate self-processing bias, results showed that people render their individual self (IS) as the primary self; specifically, the IS induced larger components linked to attention, semantic memory, and emotional value processes compared with the relational self or collective self (Chen et al., 2011; Fan et al., 2011, 2013). Contradictorily, when the self-reference task examined the memory processing of selves, the relational self became the primary self (Zhao et al., 2009; de Greck et al., 2012; Yang et al., 2013). Specifically, results showed that the relational self evoked larger late positive complex (LPC) (a late component that processes stimuli based on knowledge and experience) amplitude than the IS (de Greck et al., 2012), and activated more of the ventromedial prefrontal cortex than the collective self (Zheng et al., 2018b). Zhu et al. (2016) used gambling tasks to explore motivational hierarchy during the decision making process. They also verified the primary IS expressed when the amplitude of feedback-related negativity (FRN) was highest in the IS condition. However, there was no difference between the relational self and collective self. Another study using implicit association tasks found that Chinese people render the collective self as primary compared to the relational self (Zheng et al., 2017, 2018a). Using a priming paradigm, Wang et al. (2017) found that during the early (P2) stage, people render their IS as their primary self; their relational self as secondary; and their collective self as tertiary. However, people identify their self-hierarchy as collective self > relational self > IS during the late (P3) stage. As per the above literature, researchers have used different tasks to explore self-hierarchy with varying results.

This variant self-hierarchy emerging from the different tasks may be related to the nature of materials used in the studies. During these tasks, the self-relevant stimuli can evoke the social salience of the selves, which can induce people’s memory retrieval and emotional value attached to such information. Moreover, stimuli of these three selves tend to vary greatly in terms of familiarity and daily occurrence. The characteristics of such materials will have an impact on the processing process. For example, one’s own name can cause a subthreshold reaction (Moray, 1959). Moreover, in previous studies, using one’s name (Chen et al., 2013; Fan et al., 2013; Yang et al., 2013; Tacikowski et al., 2014; Wuyun et al., 2014) or face (Zhao et al., 2012; Fan et al., 2013; Scheepers et al., 2013; Zhu et al., 2016) as stimuli for memory or attention processing tasks inevitably led to more automatic and deeper memory recall and emotional value. The relational self, such as the name of someone close to the subject (Chen et al., 2011, 2013; Tacikowski et al., 2014; Wuyun et al., 2014) could activate memory and emotional processing. Information that represents the collective self, such as one’s hometown (Chen et al., 2011), nation (Zhao et al., 2012; Ferenczi and Marshall, 2013), or national flag (Fan et al., 2011) can also induce an emotional experience. Wang et al.’s (2017) priming paradigm proposes the necessity to compare and categorize the information related to oneself and different self-identities. Subsequently, it is essential to think and recall the identity information during this process. Thus, the use of these materials may be led to the differences of self-processing and formation of a unique self-hierarchy, which eventually led to the variant self-hierarchy during different tasks.

Since the previous paradigm could not exclude the influence of study materials, it is necessary to select a task that can exclude this influence to verify self-hierarchy. Recently, studies (Sui et al., 2012; Moradi et al., 2015, 2020; Enock et al., 2018) explored the perceptual matching task to examine self-bias when stimuli have newly formed associations with the self in order to avoid the effect caused by material’s familiarity and meaning. In this task, simple geometric shapes were arbitrarily assigned (i.e., a circle, square, or triangle) to the participant or others (e.g., someone close to the participant or a stranger) in a balanced design. Participants had to judge whether the label–shape pairings matched. During this task, researchers randomly chose neutral simple shapes to represent the self, to avoid the influence of the familiarity and meaning of self-related information to the greatest extent possible. The studies had varied results with the perceptual matching task, demonstrating a reliable overlap for perceptions of individual and collective-self advantages (Enock et al., 2018). Therefore, in this study, we adopt the perception matching task to investigate self-hierarchy in the absence of any influence of varied materials that represent the self, thus providing purer evidence for self-hierarchy.

To investigate self-hierarchy without the influence of varied materials that represent the self, we compared the perceptual advantage of the three selves, measured by perceptual matching. We aimed to reveal the situational and neural mechanism of self-hierarchy, for which we combined high temporal resolution event-related potentials (ERPs) technology to examine self-hierarchy during different processing stages. Based on previous research, we hypothesized that (1) self-hierarchy may exhibit the tendency for IS > relational self > collective self, and (2) this tendency may change with the dynamic processing stages. This line of research has important implications for broader areas of social behavior, including personal relationships and in-group and out-group behaviors, and might pave the way for a better understanding of how people can improve wellbeing by changing or at least regulating their social environment.

## Materials and Methods

### Participants

The participants were undergraduate students from Southwest University, Chongqing, initially recruited using an online screening questionnaire distributed through the campus electronic bulletin board system. Prior to data collection, we conducted a power analysis using a moderate effect size (eta squared η^2^ = 0.25) and standard power (1-β = 0.95) to determine the necessary number of participants with G*power (G*power 3; Faul et al., 2007). Assuming a within-factors repeated-measures *F*-test, the results showed that at least 36 participants were required.

The research sample included 49 participants (14 men, age range: 18–26 years, *M* = 20.04, *SD* = 1.60) who were unmarried and raised by their parents (with the mother as the primary caregiver). All of the participants were right-handed and had normal or corrected-to-normal visual acuity. After the experiment, each participant was paid 60 RMB. The research was conducted according to the ethical standards of the institutional and/or national research committee and the 1964 Helsinki Declaration and its later amendments or comparable ethical standards. Informed consent was obtained from all participants. This study was approved by the Ethics Committee of Southwest University of China (protocol code H18070 and 2018.10).

### Measures

#### Stimuli

Four of six geometric shapes (circle, square, triangle, hexagon, diamond, and octagon) were presented, with each shape being paired with information with one self construal or non-self. We asked the participants to associate each shape with information of a different self. The associations between the shapes and selves were counterbalanced across participants. Each shape (covering 3.5° × 3.5° of visual angle) and self (corresponding to 3.5° × 6.5° of visual angle) pair was presented randomly on the screen, with the shape presented approximately 4° above and below the fixation cross (0.8° × 0.8° visual angle) at the center of the screen. All the words and symbols were in white, set against a black background. The word “self (‘我自己’)” denoted the IS; “mother (‘‘我母亲’),” denoted relational self; “Chinese (‘‘中国人’’),” denoted collective self; and “stranger (‘‘陌生人’’),” denoted non-self (Cai et al., 2013; Wang et al., 2017). The stimuli were presented on a 19-inch monitor (1280 × 1024 pixels at 75 Hz). The program was run on a PC using E-prime 2.0.

#### Perceptual Matching Task

The perceptual matching task included two stages: instruction and the main task. Before the perceptual matching task, the participants were trained to associate the four shapes with different self-information types. They were asked to codename the geometric shapes as “self,” “mother,” “Chinese,” and “stranger” (lasting 60 s). An example instruction was as follows: “You are a triangle; your mother/father is a circle; Chinese is a hexagon; and a stranger is represented by an octagon.” The shapes themselves were not presented at this stage. The instructions were presented on the computer screen for as long as the participants required. Participants were informed that they could press the space key to begin the perceptual matching task once they had memorized the shape-label pairs.

They then performed the perceptual matching task. In this stage, the participants judged whether a simultaneously presented shape–label pair matched. Each trial started with the presentation of a central fixation cross for a variable time ranging from 300 to 700 ms, followed by a pairing of the shape and label at the center of the screen for 300 ms. Half of the shape–label pairs conformed to the instruction and were responded to as match trials. The remaining trials included recombination of a label with a different shape (e.g., the mother shape matched with the “self” label); these were responded to as mismatch trials. These matched and mismatched trials were randomly generated. The next frame showed a blank for a variable time ranging from 900 to 1200 ms. The participants were encouraged to respond by pressing the *F* and *J* keys as quickly and accurately as possible within this interval. Feedback was given on the screen for each trial (500 ms) only during practice, but not on the actual test (replaced by a blank for a variable time ranging from 300 to 700 ms).

Prior to the main experiment, participants repeated a practice block of 24 trials until the accuracy reached 60%. Each participant performed seven blocks of 72 experimental trials where self, mother, stranger, Chinese, and re-paired stimuli occurred equally often in a random order. Thus, each condition (self-match, self-mismatch, mother-match, mother-mismatch, Chinese-match, Chinese-mismatch, stranger-match, and stranger-mismatch) had 63 trials.

After all of the tasks, the participants completed a measurement of the degree (1–7) of closeness that they felt to their mother or father and country (adapted from Aron et al., 1992).

### Behavioral Analyses

For each participant, correct responses recorded shorter than 150 ms or longer than 1,000 ms (corresponding to approximately ± 3 *SD*s from the mean) were excluded, eliminating less than 1% of the trials overall. For analyzing response times (RTs), we only computed the correct responses.

Previous studies (Enock et al., 2018; Desebrock and Spence, 2021) propose a signal detection approach calculated using a sensitivity index (d prime, d’) was instead of the traditional accuracy. Hits were coded as correct responses for matching conditions and false alarms were coded as incorrect responses for mismatching conditions.

### Electroencephalogram Recording and Analyses

We recorded brain electrical activity from 64 scalp sites using tin electrodes mounted in an elastic cap (Brain Products GmbH, Gilching, Germany), with the reference electrodes placed on the fronto-central aspect (FCz) and a ground electrode on the medial frontal aspect (AFz). The vertical electrooculogram (EOG) was recorded with an electrode placed infraorbitally in the right eye. All inter-electrode impedances were maintained at below 5 KΩ.

We performed data processing using MATLAB R2014a using the EEGLAB toolbox 14.1.1 b. Individual and grand ERPs averages were created for the individual, relational, collective, and non-self stimuli, and the resulting grand averages were based on the correct trials. We first down sampled the data from 1,000 to 256 Hz and performed high- and low-pass filtering at 0.1 and 45 Hz, respectively. We selected the left and right mastoids as the reference sites. Data were epoched from 200 ms prior to stimulus onset to 1,000 ms after presentation and were baseline-corrected to the pre-stimulus interval. Trials were excluded if they included EOG artifacts (ocular movements and eye blinks). We excluded artifacts arising from amplifier clipping, bursts of electromyographic activity, or peak-to-peak deflections exceeding ± 80 μV from averaging prior to conducting independent component analysis (ICA). We found no differences in trial counts between the different selves or between the matched and mismatched conditions. The components, including EOG artifacts and head movement, were removed from the ICA results after visual inspection. Based on previous studies (Wang et al., 2017; Li et al., 2019) and the purpose of this study, we identified the topographical distribution of the grand-averaged ERPs activities, ERPs components, and their time epochs as follows: P2 (180–240 ms), N2 (280–340 ms), P3 (350–420 ms), and LPC (500–800 ms). We selected the following electrode sites: frontal (F3, Fz, F4), frontal-central (FC3, FCz, FC4), central (C3, Cz, C4), central-parietal (CP3, CPz, CP4), and parietal (P3, Pz, P4). Since this study aimed to explore the self-hierarchy, we could only determine the shape that activated the corresponding self label under the matching condition; thus, we focused only on the differences in ERPs during the matching condition (Wang et al., 2017). Therefore, repeated-measures analyses of variance [4 (self: IS IS; relational self, RS; collective self, collective selves (CS); and non-self, NS) × 5 (electrode site: frontal, frontal-central, central, central-parietal, and parietal)] were conducted on the amplitudes of P2, N2, P3, and LPC, with two within-subjects factors. All analyses were conducted using IBM SPSS Statistics for Windows, Version 22.0. The *p*-values were adjusted for sphericity using the Greenhouse–Geisser method. *Post-hoc t*-tests with Bonferroni adjustments were used for multiple comparisons. We conducted outlier analyses on EEG data using ± 3 *SD*s and found that all EEG data had ± 3 *SD*s. Therefore, all EEG data were included in the analyses.

## Results

### Questionnaire Results

On the seven-point Likert scale for measuring closeness between self and mother/father (1 = *no overlap*; 7 = *full overlap*), the rating scores ranged from 1 to 7 (*M* = 4.26, *SD* = 1.74). Meanwhile, scores for the closeness between self and country ranged from 1 to 7 (*M* = 3.60, *SD* = 1.88).

### Behavioral Results

**FIGURE 1:**
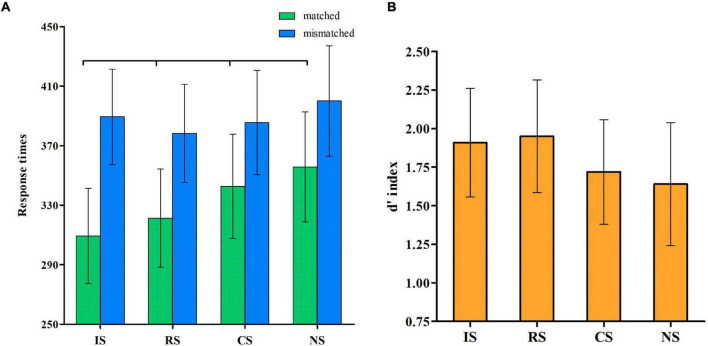
Self-association effect. **(A)** Shows the mean response times of the matched and mismatched pairs for the different selves. **(B)** Shows the *d*’ index for the different selves.

Table 1 shows the details of the RTs under the different conditions. We used 4 × 2 analyses of variance with the two within-subjects variables of the selves and matching judgment. The analysis results of the RTs (Figure 1A) showed the significant main effects of self, *F*_(3, 46)_ = 19.98, *p* < 0.001, η^2^ = 0.56, observed power = 1.00, and matching condition, *F*_(1, 48)_ = 195.27, *p* < 0.001, η^2^ = 0.80, observed power = 1.00. Results for RT also showed that the interaction between the self and match variables was significant, *F*_(3, 46)_ = 7.32, *p* < 0.001, η^2^ = 0.32, observed power = 0.98. A simple-effect analysis showed that in match trials, the RTs during IS were faster than those during the RS, CS, and NS conditions, all *p*s < 0.05. Moreover, the RTs during the RS condition were significantly faster compared with the CS and NS conditions, all *p*s < 0.5, and those during the CS condition were significantly faster compared with the NS condition. However, in mismatch trials, the RT during NS condition was the longest, all *p*s < 0.05.

**TABLE 1:** Mean reaction times for the variables of self and matching condition.

		IS		RS		CS		NS	

		M	SD	M	SD	M	SD	M	SD
RT	Match	309.36	98.72	321.29	101.58	342.63	110.11	355.71	118.18
	Mismatch	389.39	128.74	378.19	118.53	390.39	123.28	400.02	124.34

*RT, reaction time; IS, individual self; RS, relational self; CS, collective self; NS, non-self; M, mean; SD, standard deviation.*

Next, we compared the *d*’ values for the IS, RS, CS, and NS (Figure 1B). The results revealed a significant main effect of the self, *F*_(3, 46)_ = 21.27, *p* < 0.001, η^2^ = 0.58, observed power = 1.00. Upon further analyzing the main effect, the *post-hoc t*-test did not reveal any significant difference between the *d*’ during the IS and RS conditions, but both were higher than those during the CS (*t* = 0.19, *t* = 0.23, respectively) and NS (*t* = 0.27, *t* = 0.31, respectively) conditions, all *p*s < 0.001.

### Event-Related Potentials

**FIGURE 2:**
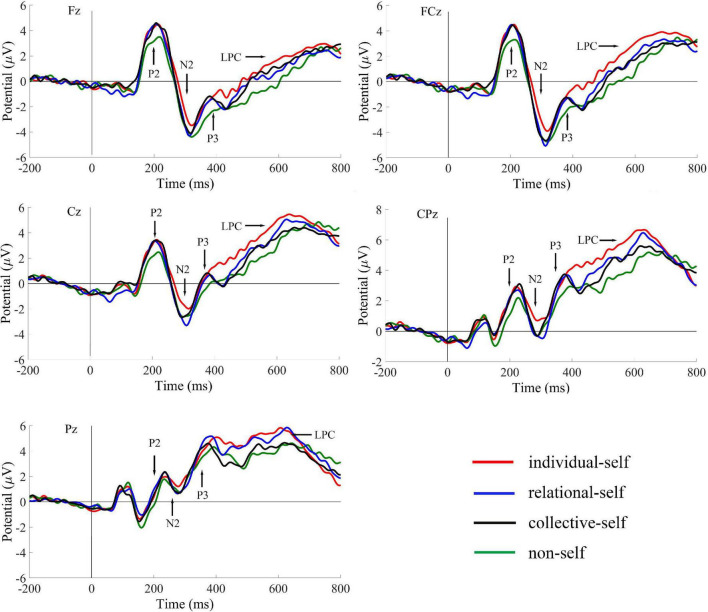
Grand-averaged ERPs for P2, N2, P3, and LPC of the individual self, relational self, collective self, and non-self during the matched condition.

**FIGURE 3:**
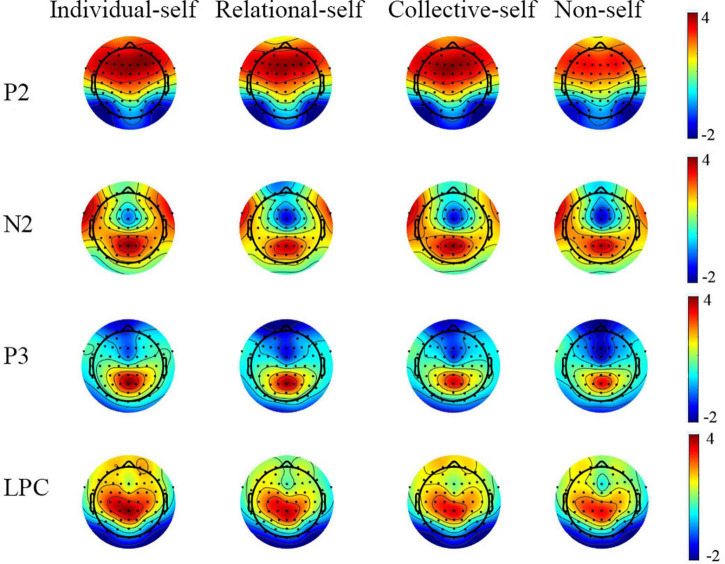
Topography plots of P2, N2, P3, and LPC for the individual self, relational self, collective self, and non-self during the matched condition.

Only the average ERPs during matching conditions were compared. The grand-averaged ERPs of P2, N2, P3, and LPC at Fz, FCz, Cz, CPz, and Pz in the matched condition for IS, RS, CS, and NS are shown in Table 2 and Figure 2; topography plots are presented in Figure 3.

**TABLE 2:** Mean amplitudes of P2, N2, P3, and LPC of the self variables in the matched condition.

		Frontal	Frontal-central	Central	Central-parietal	Parietal
N2	IS	−1.66 ± 3.48	−2.06 ± 3.55	−0.96 ± 3.35	0.44 ± 3.23	1.07 ± 3.10
	RS	−2.57 ± 3.76	−2.86 ± 3.96	−1.84 ± 3.76	−0.26 ± 3.58	0.88 ± 3.32
	CS	−2.46 ± 3.85	−2.67 ± 3.96	−1.52 ± 3.69	0.16 ± 3.45	1.12 ± 3.29
	NS	−2.63 ± 3.66	−2.78 ± 3.65	−1.62 ± 3.36	−0.06 ± 3.28	0.86 ± 3.48
P2	IS	3.73 ± 3.32	3.42 ± 3.26	2.51 ± 3.12	1.44 ± 2.61	−0.01 ± 2.44
	RS	3.54 ± 3.25	3.26 ± 3.36	2.36 ± 3.16	1.37 ± 2.77	0.28 ± 2.78
	CS	3.62 ± 3.5	3.4 ± 3.57	2.47 ± 3.29	1.41 ± 2.79	0.07 ± 2.57
	NS	2.91 ± 3.21	2.63 ± 3.22	1.73 ± 3.16	0.74 ± 2.84	−0.39 ± 2.56
P3	IS	−0.64 ± 3.38	−0.24 ± 3.28	1.05 ± 3.13	2.53 ± 3.15	2.92 ± 3.15
	RS	−1.29 ± 3.76	−0.89 ± 3.84	0.43 ± 3.9	2.17 ± 4.01	2.98 ± 3.7
	CS	−1.22 ± 3.75	−0.88 ± 3.51	0.35 ± 3.34	1.97 ± 3.22	2.38 ± 3.37
	NS	−1.56 ± 3.05	−1.16 ± 2.82	0.18 ± 2.79	1.74 ± 2.9	2.3 ± 3.37
LPC	IS	2.46 ± 3.45	3.23 ± 3.43	4.24 ± 3.22	4.33 ± 2.88	2.86 ± 2.52
	RS	1.7 ± 3.37	2.53 ± 3.52	3.59 ± 3.5	3.98 ± 3.28	2.9 ± 2.82
	CS	1.77 ± 3.58	2.54 ± 3.7	3.33 ± 3.49	3.63 ± 3.09	2.39 ± 2.97
	NS	1.52 ± 3.38	2.34 ± 3.19	3.35 ± 3.29	3.63 ± 3.08	2.54 ± 3.02

*IS, individual self; RS, relational self; CS, collective self; NS, non-self.*

#### P2

Repeated-measures analysis of variance (ANOVA) for self × electrode site showed the main effect of self, *F*_(3, 46)_ = 4.43, *p* < 0.01, η_*p*_^2^ = 0.22, observed power = 0.85, with the *post-hoc t*-test showing that P2 amplitudes during the IS (*t* = 0.69) and CS (*t* = 0.67) conditions were significantly greater than those during the NS condition (all *p*s < 0.05). Moreover, the P2 amplitudes during the RS condition showed marginal significance than during the NS condition, *t* = 0.64, *p* = 0.09. However, we found no difference among IS, RS, and CS. The results also showed a main effect of electrode site, *F*_(4, 45)_ = 19.53, *p* < 0.001, η_*p*_^2^ = 0.64, observed power = 1.00. The *post-hoc t*-test showed that P2 amplitudes were greater in the frontal and frontal-central regions, and the magnitude of P2 amplitudes was as follows: frontal = frontal-central > central > central-parietal > parietal, all *p*s < 0.05.

#### N2

Repeated-measures ANOVA of the self × electrode site variables showed the main effects of self, *F*_(3, 46)_ = 3.83, *p* = 0.01, η_*p*_^2^ = 0.20, observed power = 0.79, and electrode site, *F*_(4, 45)_ = 25.93, *p* < 0.001, η_*p*_^2^ = 0.70, observed power = 1.00. For the interaction of self and electrode site, *F*_(12, 37)_ = 2.72, *p* = 0.01, η_*p*_^2^ = 0.47, observed power = 0.94, a simple-effect analysis showed that N2 amplitudes in the IS condition were significantly smaller than those in the NS (*t* = 0.97, *t* = 0.72), RS (*t* = 0.90, *t* = 0.80), and CS (*t* = 0.80, *t* = 0.62) conditions in the frontal and frontal-central regions (all *p*s < 0.05). However, in the central region, N2 amplitudes in the IS condition were significantly smaller than those in the RS condition (*t* = 0.71, *p* < 0.05). No significant differences were found between the RS and CS condition.

#### P3

The results for P3 showed the main effects of self, *F*_(3, 46)_ = 4.53, *p* < 0.01, η_*p*_^2^ = 0.23, observed power = 0.86, and electrode site, *F*_(4, 45)_ = 32.07, *p* < 0.001, η_*p*_^2^ = 0.74, observed power = 1.00. For the interaction of the self and electrode site variables, *F*_(12, 37)_ = 2.31, *p* = 0.01, η_*p*_^2^ = 0.33, observed power = 0.74, a simple-effect analysis showed that P3 amplitudes in the IS condition were significantly greater than those in the NS (*t* = 0.92, *t* = 0.92, *t* = 0.87), RS (*t* = 0.65, *t* = 0.65, *t* = 0.63), and CS (*t* = 0.57, *t* = 0.64, *t* = 0.70) conditions in the frontal, frontal-central, and central regions (all *p*s < 0.05). However, in the parietal region, P3 amplitudes in the IS and RS conditions were significantly greater than those in the CS (*t* = 0.54, *t* = 0.60) and NS (*t* = 0.62, *t* = 0.68) conditions (all *p*s < 0.05).

#### Late Positive Complex

Repeated-measures ANOVA of the self × electrode site variables for LPC showed the main effects of self, *F*_(3, 46)_ = 3.26, *p* = 0.03, η_*p*_^2^ = 0.18, observed power = 0.71, and electrode site, *F*_(4, 45)_ = 25.89, *p* < 0.001, η_*p*_^2^ = 0.70, observed power = 1.00. For the interaction of self and electrode site, *F*_(12, 37)_ = 2.20, *p* = 0.01, η_*p*_^2^ = 0.30, observed power = 0.73, a simple-effect analysis showed that LPC amplitudes were significantly greater in the IS condition than those in the NS (*t* = 0.94, *t* = 0.90, *t* = 0.90), RS (*t* = 0.76, *t* = 0.71, *t* = 0.65), and CS (*t* = 0.68, *t* = 0.70, *t* = 0.91) conditions in the frontal, frontal-central, and central regions (all *p*s < 0.05). However, in the parietal region, LPC amplitudes in the RS condition were significantly greater than those in the CS condition, *t* = 0.51, *p* < 0.05.

## Discussion

The present work sought to examine self-hierarchy without the influence of varied materials which represent the self, using a simple perceptual matching task. The behavioral results showed that people recognize self-related information faster and more accurately compared to non-self-related information. Using the high temporal resolution of ERPs, our study showed that self-hierarchy changed based on the processing stage. We found a weaker advantage for associations with the relational and CS compared with the IS, especially at the late processing stage. People recognized information associated with the IS faster than they did with the relational and CS, according to the stronger P3 and LPC amplitudes and the smaller N2 amplitudes evoked during IS conditions. Moreover, the collective self showed a weaker advantage than the relational self, with a longer reaction time, lower d prime, and weaker P3 and LPC amplitudes in the parietal region. Overall, the self-hierarchy during simple perceptual processing manifested as IS > relational self > collective self at the late processing stage, but as IS = relational self = collective self at the early processing stage.

After excluding the social context and familiarity with different self-related stimuli, we confirmed the priority for the IS in self-hierarchy, reflected in the faster RT, smaller N2, stronger P3, and LPC amplitudes. The behavioral results were consistent with the ERP results, with people reporting faster RTs for IS than CS and NS. Based on previous studies, N2 typically represents the frontier between automatic and controlled processing phases and is associated with a conversion mechanism for attention, while P3 has mainly been associated with access to attention, semantic memory, and emotional value of stimuli (Tacikowski and Nowicka, 2010; Tacikowski et al., 2014; Constable et al., 2019). The smaller N2 amplitudes suggested that people process IS more automatically, and the conversion between automatic and controlled processing was more flexible. Greater P3 amplitudes suggested that people engaged more attention, memory, and emotional resources when processing IS. The combination of N2 and P3 indicated that when processing IS, people evoked various cognitive resources more quickly. Taken together, faster RT, smaller N2, and larger P3 indicated that IS can be noticed and processed faster. Our results were consistent with the results in previous research using self-relevant information (Cunningham et al., 2008; Chen et al., 2011; Sui et al., 2013; Tacikowski et al., 2014). Chen et al. (2013) used one’s forename as the IS and one’s surname as the collective self to measure the IS as the primary self. They found that smaller N2 and larger P3 amplitudes are elicited by IS-relevant stimuli as compared with those by elicited collective self-relevant stimuli. Sui et al. (2013) used participants’ own and friend’s faces as IS and RS, respectively, and found that RS elicited larger N2 amplitudes than IS. Liu et al. (2013) also revealed that IS elicited higher P3 than RS did (Liu et al., 2013). Moreover, similar to a previous study (Wang et al., 2017), we found that the IS evokes a larger LPC compared with the relational and CS. Like P3, LPC is also a late component that process stimuli based on knowledge and experience (Song et al., 2019). Since we employed the perception matching task, our results could provide further evidence that even for simple shapes that did not involve emotions or meaning, the information is more easily retrieved and larger attentional and cognitive resources are recruited, if it establishes a temporary connection with self, especially the IS. The IS showed a high self-hierarchy even without the semantic memory and emotional value of self-related information, such as one’s name or face, during simple perceptual processing.

In line with previous results (Wang et al., 2017), the relational self evoked greater P3 and LPC amplitudes compared with the collective self. Enhanced P3 in response to the relational self suggests that this self type recruits a larger amount of attentional and cognitive resources and evokes enhanced emotional/motivational responses compared with the collective self. Therefore, larger P3 amplitudes for the relational self vs. the collective self further demonstrated that the relational self is more emotionally and motivationally engaging compared with the collective self in the self-concept. The collective self lags behind the relational self and is less functional in fulfilling one’s teleological ideal (Nehrlich et al., 2019), which can explain the stronger perceptual advantage for the relational self compared with the collective self. In our study, the RS > CS tendency only occurred in the parietal region. Inconsistently, Zheng found that the RS generates stronger medial prefrontal cortex (MPFC) activity compared with the CS (Zheng et al., 2018b). Neuroimaging studies suggest that some parts of the “social brain,” including the medial part of the prefrontal cortex (Sui et al., 2013), might play a role in the enhanced processing of stimuli that are emotionally salient and related to the self. Previous fMRI studies indicated that RS shared an overlapping neuro mechanism-MPFC-with IS, but not with CS (Zhu et al., 2007; Zheng et al., 2017). However, in the present study we did not find any difference in ERPs between RS and CS in the frontal regions. This inconsistent in results may have arisen due to the replacement of specific information related to the relational and CS with new and temporary associations; accordingly, there was no participation of the “social brain” in perception processing.

The above results confirm that the self-hierarchy showed a trend of individual > relational > collective in the late processing stage after 280 ms. However, at the early stage, P2 components did not show any difference between the three selves. This result may be due to the fact that during the perceptual matching task, participants were only required to make a choice about whether the label-shape matched, without activating deep thinking about their individual, relational, and collective identity. P2 represents a subjacent neural and autonomic processing stage (Chen et al., 2013); thus, the simple shape of newly established connections with the three selves did not cause early differences during people’s perceptual processing in our study. All the three selves emerge into people’s attention in the early stage of processing quickly and automatically. The diverse results of N2, P3, LPC at the late stage (after 280 ms) and P2 at the early stage (before 280 ms) verified our hypothesis 2 indicating that self-hierarchy may change with the dynamic processing stages. Inconsistent with our results, a study found a primacy of the collective self vs. the IS and relational self when using self-relevant information and asked participants to make subjective judgements on these stimuli (Wang et al., 2017). Researchers also found that at the early stage, especially during implicit processing, the sense of belonging to the group and self-identity as a group member is prominent (Zheng et al., 2018a). The difference in results may be due to our perception matching paradigm that excluded the influence of self-related information.

The results of our study indicate that the self-hierarchy could also pervade perceptual processing even without meaningful information. It is important to examine the self-hierarchy rule outside of the influence of semantic memory and emotional value of self-related information. Self-hierarchy can predict the differences of the individual, relational, and collective self not only in recognition, attitude, motivation, and other social behaviors, but also in simple perception processing. This provides further evidence for the tripartite model of self-construal theory. Researchers found that people can also rapidly tag neutral actions with a personal association, thereby making their behavior toward the self more efficient (Frings and Wentura, 2014; Moradi et al., 2018; Desebrock and Spence, 2021). Combined with our finding, all three selves have a self-prioritization effect, and can be tagged with neutral actions, which would fit the idea of an organism attuned to self-survival at multiple processing levels.

Some limitations of this study should be noted. First, our findings may not be generalizable to non-Chinese samples. Since the self-hierarchy was contextual, it may be differ under varied cultural backgrounds (Han and Ma, 2014; Huang et al., 2014; Mamat et al., 2014). Cross-cultural neuroscience research has confirmed that compared with people in the West, East Asians may have more socially embedded conceptualizations of the relational self and IS (Chiao et al., 2013; Han et al., 2016). Participants from different cultural backgrounds should be included in future studies. Second, we only used the words “mother” and “China” for measuring the relational and CS. Although our study did not involve the processing mechanism of self-related information directly, it is unclear whether the self-hierarchy pattern would extend to other relationships (e.g., friendship, romantic partner) or in-groups (e.g., fan clubs, ethnic groups).

## Conclusion

Our behavioral and ERPs findings suggested that self-hierarchy during simple perceptual processing manifested as IS > relational self > collective self at the late processing stage. At the early stage, the hierarchy manifested as IS = relational self = collective self. In the context of a Chinese collectivist cultural background, self-hierarchy was shown to vary according to the processing stage, even without meaningful information that evoked social salience during recognition and motivation processing. This result provides direct evidence of the situational nature of self-constructs.

## Data Availability Statement

The raw data supporting the conclusions of this article will be made available by the authors, without undue reservation.

## Ethics Statement

This study was conducted according to the guidelines of the Declaration of Helsinki, and approved by the Institutional Review Board of Southwest University (protocol code H18070). The patients/participants provided their written informed consent to participate in this study.

## Author Contributions

YZ and ZX: conceptualization and funding acquisition. YZ and XZ: methodology. YZ and YL: data collection. YZ: writing—original draft preparation. YZ, ZX, and YL: writing—review and editing. All authors have read and agreed to the published version of the manuscript.

## Conflict of Interest

The authors declare that the research was conducted in the absence of any commercial or financial relationships that could be construed as a potential conflict of interest.

## Publisher’s Note

All claims expressed in this article are solely those of the authors and do not necessarily represent those of their affiliated organizations, or those of the publisher, the editors and the reviewers. Any product that may be evaluated in this article, or claim that may be made by its manufacturer, is not guaranteed or endorsed by the publisher.
